# Exploring an Infectious Disease Service Pathway for Unaccompanied Asylum‐Seeking Children: An Audit and Retrospective Analysis

**DOI:** 10.1155/ijpe/3622069

**Published:** 2026-07-15

**Authors:** Holly Tarn, Michelle Bond

**Affiliations:** ^1^ Department of Academic Research, Brighton and Sussex Medical School, Brighton, Sussex, UK, bsms.ac.uk; ^2^ Department of Looked After Children, Sussex Community NHS Foundation Trust, Brighton and Hove Children′s Council Services, Brighton, Sussex, UK

**Keywords:** asylum-seeker, chlamydia, hepatitis, infection, syphilis, tuberculosis, unaccompanied

## Abstract

Unaccompanied asylum‐seeking children (UASC) experience a high burden of infectious diseases (IDs) and are a highly vulnerable population group with significant mental, physical, social and developmental needs. However, currently in the United Kingdom, there is no framework regarding how to implement ID screening guidance for UASC, leading to a wide variation in outcomes. This project is aimed at exploring the ID pathway for UASC within a single South‐East UK local authority, spanning two trusts—including one with a ‘one‐stop’ clinic—to inform service improvement recommendations. The study was conducted by retrospective case note review of routinely collected, anonymised patient data from all UASC under the care of the local authority over a 1‐year timeframe. The study design consisted of (a) an audit of UASC ID referrals for all children in the local authority against RCPCH guidance, (b) analysis of attendance rates and (c) analysis of reasons for noncompletion. Thirty‐seven patients were analysed (local trust *n* = 30, neighbouring trusts *n* = 7). The pathway′s success varied across different aspects. Tuberculosis (TB) and blood‐borne virus (BBV) testing showed notably higher referral and attendance rates compared to other single‐centre studies. However, gaps in the process were evident in areas such as testing for schistosomiasis and strongyloidiasis. Additionally, the appointment completion rate varied across ID services, highlighting some themes in low attendance, in particular a low attendance rate for those ‘out of area’. Our key recommendation is the undertaking of further research to inform the development of a more robust pathway for UASC in the region, with consideration of a county‐wide pathway.

**Key Messages**

• Standardising services for unaccompanied asylum‐seeking children (UASC), supported by best practice guidance, can reduce practice variation and related health disparities.
• Children referred out of area show lower rates of infectious disease (ID) screening.
• Future research should focus on streamlined care pathways to reduce wait times and appointments, improving attendance and timely ID treatment.

## 1. Introduction

The number of UASC in the United Kingdom has been growing over the past decade, with 1371 asylum applications made in the year ending June 2023 [1]. UASC are a highly vulnerable population group with significant mental, physical, social and developmental needs [2, 3]. This includes a high burden of IDs [4]. It is therefore important to analyse ID care pathways to guarantee optimal care for this vulnerable demographic.

In the United Kingdom, UASC are cared for by the state as children in care (CICs) within the local authority in which they first present. A local authority is a government body responsible for delivering local services within a defined geographic area. All CICs are required to undergo a statutory initial health assessment (IHA), led by a paediatrician, within 20 days of entering care [5]. Following the IHA, a health report and care plan are produced, including referrals to relevant health services such as ID services [3]. General practitioners (GPs) in the United Kingdom provide routine primary care for this cohort but do not hold responsibility for coordinating screening; testing and vaccinations are undertaken as directed by the IHA team.

Following the IHA, referrals to the next ID appointments are made, and the services available vary across the United Kingdom. Pathways differ depending on proximity to specific NHS trusts, which are organisational units responsible for delivering healthcare services. These trusts are geographically distinct from local authority boundaries.

This study examines two groups of children within a single local authority who are served by different NHS trusts. Children residing near a large urban centre follow a simplified pathway involving two referrals. First, they are referred to a paediatric ‘one‐stop’ ID clinic at a local acute hospital. Such clinics aim to minimise the number of appointments by consolidating testing; however, at the time of data collection, this clinic screened only for tuberculosis (TB), hepatitis B, hepatitis C and human immunodeficiency virus (HIV) and did not routinely include testing for syphilis, strongyloidiasis or schistosomiasis. Second, children are referred to their GP to initiate vaccination schedules.

In contrast, children placed further from this clinic fall under a smaller healthcare trust, and they follow a complex pathway involving multiple referrals. Screening for TB and blood‐borne viruses may occur across different settings, including primary care and hospital‐based adult respiratory services. Pathways for sexual health screening are not clearly defined, whilst vaccinations are delivered through primary care. In many cases, three or more referrals are required to complete recommended screening.

In order to support paediatricians′ care for UASC, the Royal College of Paediatric and Child Health (RCPCH) has written national guidance [6]. The key guidance regarding IDs and immunisations has been outlined below (see Figure 1a).

Figure 1(a) Royal College of Paediatric and Child Health guidance: ‘Children and Young People Seeking Asylum and Refugees ‐ Guidance for Paediatricians’ [6]. (b) Guidance basis, eligibility criteria and target metric for audit.

**Figure:**
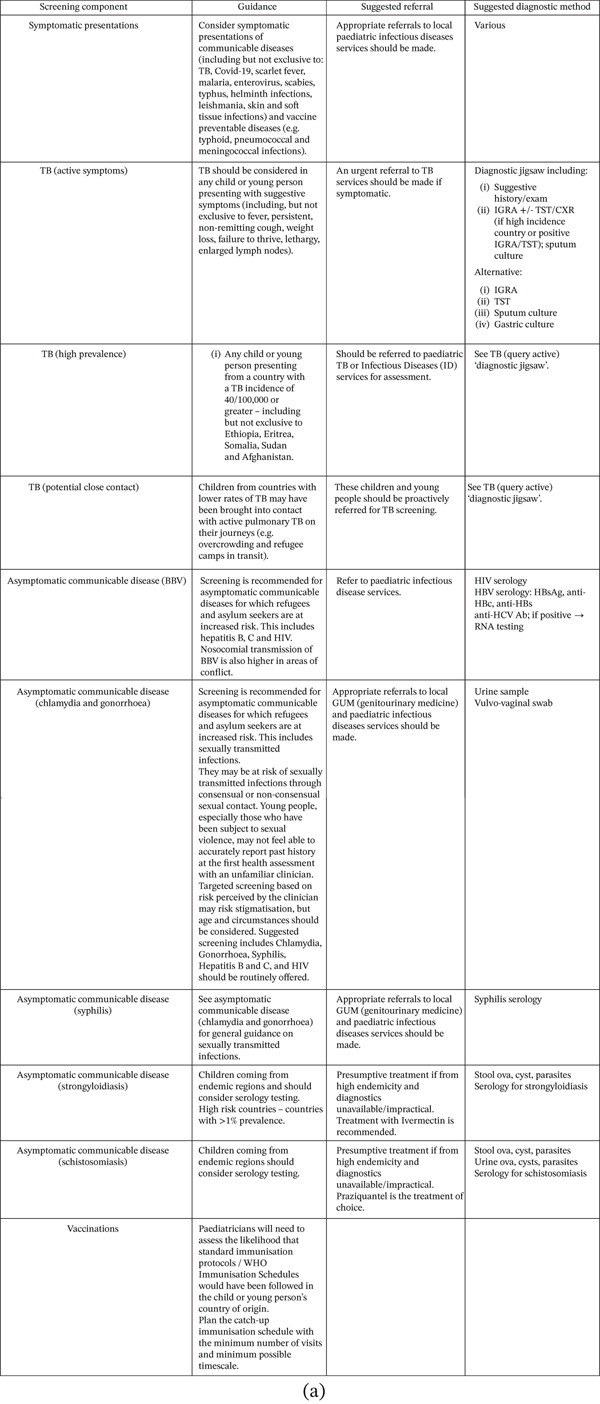
(a)

**Figure:**
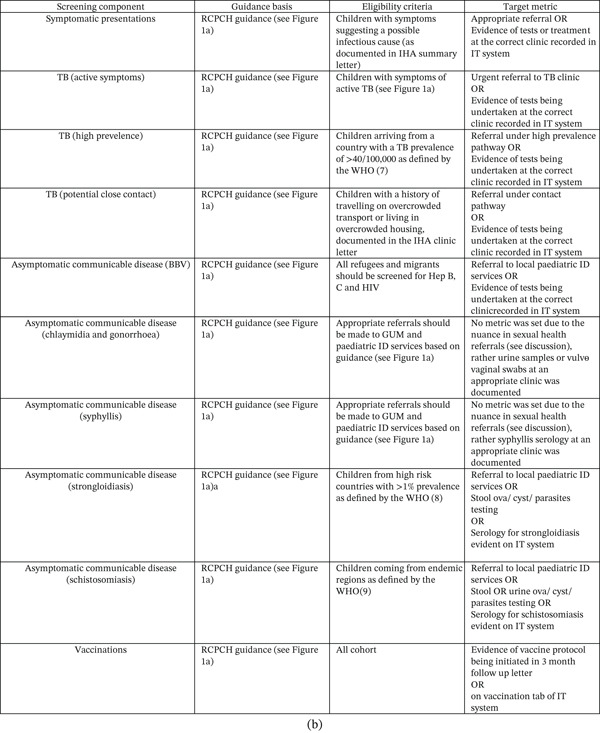
(b)

Both nationally and locally, there is a lack of empirical data analysing the efficacy of pathways for UASC ID services. Here, we aim to audit all IHAs undertaken for UASC in a single local authority over a 1‐year timeframe to assess adherence to RCPCH guidance and to analyse appointment outcomes and reasons for completion to build broad hypotheses around the barriers that UASC may face when accessing ID services in the region. We aim to use this data to develop service improvement recommendations and to aid further research.

## 2. Methods

Data was collected via retrospective case note analysis of 37 children under the care of the local authority who had their IHA between 01.08.2022 and 01.08.2023. The inclusion criteria included age < 18, patient records available and patient confirmed as a UASC. Then, 100% of participants (*n* = 37) met the inclusion criteria. A data collection tool was self‐designed using Microsoft Excel, and data was anonymised prior to input.

Each ID referral was documented as ‘made in line with guidance’ or not. This was unique for each referral according to the guidance (see Figure 1a,b). For example, for BBV and vaccinations, it is recommended that all UASC are referred (unless there is evidence of a full vaccination schedule, which was not the case for any of this cohort); therefore, the target referral rate is 100%. However, for sexually transmitted infections (STIs), the referral recommendations are more nuanced (see Figure 1 and section 3), and therefore, it was documented only whether a referral was made or not, rather than whether the referral was ‘made in line with guidance’. The guidance was based solely on the RCPCH guidance. Figure 1b outlines the eligibility criteria and target metric for this audit.

In terms of missing data, if no referral letter was identified in the IT system, laboratory results and follow‐up letters were reviewed. Completion of laboratory tests at the appropriate clinic was taken as evidence that a referral had occurred. Cases with no referral letter or laboratory evidence were classified as ‘no referral’. Where a referral letter was present, but no laboratory results or follow‐up documentation was found, this was classified as ‘referral made but not attended’.

Ethical approval was waived for this research project, as it was deemed unnecessary for an audit.

### 2.1. Findings

#### 2.1.1. Demographics

**Figure 2:**
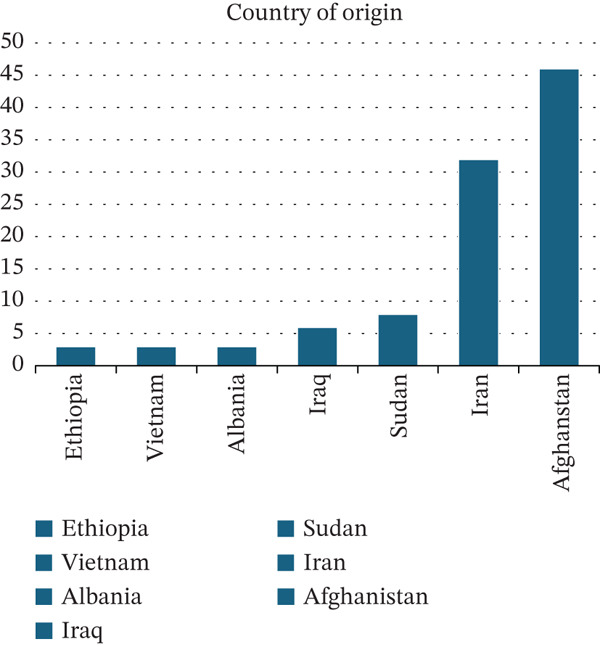
The countries of origin of the cohort.

**Figure 3:**
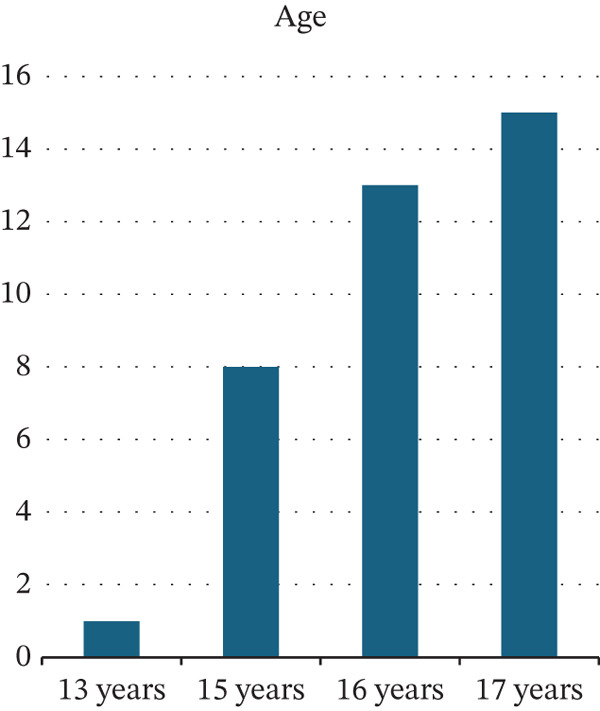
The age distribution of the cohort.

• Thirty‐seven patients were included; 36/37 (97%) were male.
• Countries of origin: Afghanistan (17/37, 46%), Iran (12/37, 32%), Sudan (3/37, 8%), Iraq (2/37, 6%), Albania (1/37, 3%), Ethiopia (1/37, 3%) and Vietnam (1/37, 3%) (Figure 2).
• Ages ranged from 13 to 17 (mean = 16.1) (Figure 3).
• 30/37 (81%) had their IHA at a ‘one‐stop’ clinic; 7/37 (19%) at neighbouring trusts.

#### 2.1.2. TB and BBV

**Figure 4:**
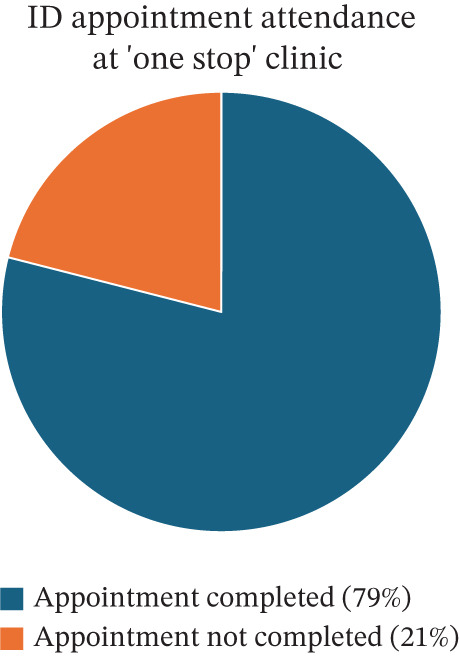
Rates of referrals and attendance for the ‘one‐stop’ clinic at local acute hospital.

**Figure 5:**
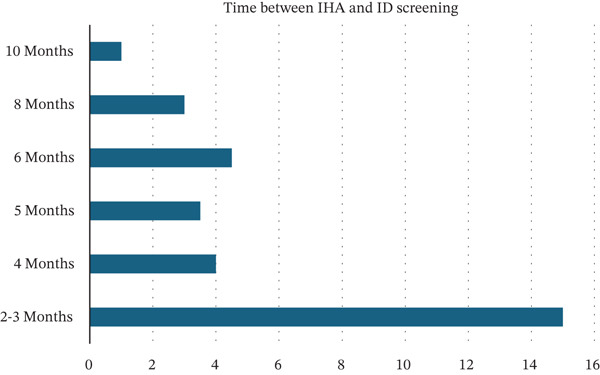
Wait times between IHA and ID appointment at local acute hospital ‘one stop’ clinic.

**Figure 6:**
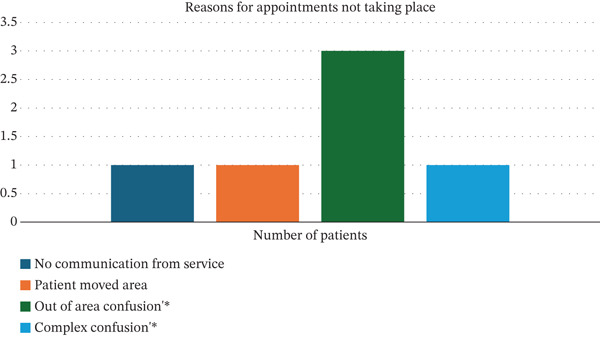
Reasons for appointments at ‘one‐stop’ clinic not taking place.  ^∗^See section 3 for a description of ‘out‐of‐area confusion’ and ‘complex confusion’.

• All 30/30 (100%) ‘one‐stop’ patients were referred for TB/BBV screening in line with guidance.
• 23/30 (77%) attended (Figure 4).
• Median wait: 3 months (range: 3–10); one delay due to an NHS number error (Figure 5).
• 15/30 (50%) were from high TB prevalence countries (Afghanistan, Sudan, Ethiopia and Vietnam). All were referred; 10/15 (66%) attended.
• The other 15/30 (50%) were from lower prevalence areas but came through the contact risk pathway; 14/15 (92%) were referred, and 13/14 (93%) of those attended.
• No patients had TB symptoms or positive results.
• Missed appointments were due to lack of communication (1/6), patient moved (1/6), ‘out‐of‐area’ confusion (3/6) and complex situations (1/6) (Figure 6).

#### 2.1.3. Chlamydia, Gonorrhoea and Syphilis

**Figure 7:**
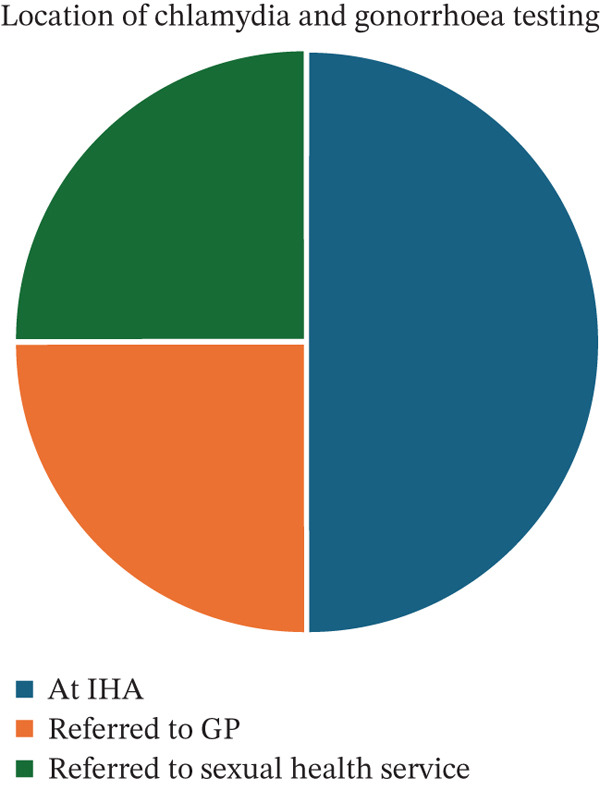
Location of chlamydia and gonorrhoea testing.

• 4/30 (13%) had chlamydia/gonorrhoea testing; none were tested for syphilis.
• 1/4 (25%) tested positive for chlamydia.
• Those that had chlamydia/gonorrhoea testing were sent to the GP (1/4, 25%), referred to a sexual health service (1/4, 25%) or had the testing at the IHA (2/4, 25%) (Figure 7).

#### 2.1.4. Vaccinations

• 28/29 (96%) were referred for vaccinations.
• Of those referred, 12/28 (42%) attended one or more sessions, and 16/28 (57%) did not.
• Reasons: foster carers did not book (7/16, 43%), no GP communication (4/16, 25%), out‐of‐area confusion (2/16, 12%), hesitancy (1/16, 6%), translator issues (1/16, 6%) and did not attend (1/16, 6%).
• Median time for attended appointments: 4 months.

#### 2.1.5. Symptomatic Presentations

• 8/30 (26%) presented with symptoms: scabies (4/8), digestive issues (2/8), infected wound (1/8) and bite (1/8).
• All were referred onward. One *Helicobacter pylori* case was detected via GP.

#### 2.1.6. Schistosomiasis, Strongyloidiasis and Other Soil‐Transmitted Helminthiases

• 17/30 (56%) were from countries endemic for strongyloidiasis/helminths, but none were referred.
• 4/30 (13%) were from schistosomiasis‐endemic regions; none were referred.

#### 2.1.7. Other Barriers

• Interpreter issues were noted in 8/30 (26%).
• Vaccine hesitancy/needle phobia in 7/30 (23%).
• Communication confusion in 13/30 (43%).
• 5/30 (16%) were referred out of area; 3/5 (60%) of these faced confusion leading to missed appointments.

### 2.2. Neighbouring Trusts—Key Findings

Percentages have been avoided due to the small sample size in the neighbouring trusts and to prevent direct comparisons between the two trusts (see section 3).

• BBV referrals:
  o. 5/7 participants were referred.
  o. Three attended; two tested positive for hepatitis B.
  o. 4/7 had no BBV testing.
  o. Missed appointment reasons:
    ▪ Patient unsure/no results [2].
    ▪ Not booked by social worker [2].
• TB testing:
  o. 5/7 were from high TB prevalence areas.
  o. Four were referred; two attended, and both tested positive for latent TB.
  o. Missed appointment reasons:
    ▪ Patient unsure/no results [1].
    ▪ Not booked by patient/carer/social worker [1].
• TB close contacts:
  o. 2/7 had potential TB contact during travel.
  o. One was referred but did not attend; not booked by patient/carer/social worker.
• STI testing:
  o. 0/7 were referred for or tested for chlamydia, gonorrhoea or syphilis.
• Vaccinations:
  o. 4/7 were referred for vaccinations.
  o. One declined referral; two had no referral made.
  o. 0/7 attended their vaccination appointments.
  o. Missed appointment reasons:
    ▪ No communication from GP [2].
    ▪ Not booked by patient/carer/social worker [2].
• Symptomatic presentation:
  o. 0/7 had a symptomatic presentation at screening.
• Parasitic infections:
  o. 4/7 were from countries endemic for strongyloidiasis or other soil‐transmitted helminthiases.
  o. 0/4 were referred for testing.
  o. 0/7 were from schistosomiasis‐endemic countries.
• Communication barriers and additional factors:
  o. Interpreter issues interfered with scheduling in 2/7 cases.
  o. Needle phobia/vaccine hesitancy was noted in 3/7 cases.
  o. In all seven cases, confusion between practitioners and patients was documented regarding appointment timing or completion.

## 3. Discussion

In this UASC cohort, the pathway showed positive outcomes in some areas, particularly TB and BBV testing, where referral and attendance rates were higher than in other single‐centre studies [7]. However, there were notable gaps, including low testing for syphilis, schistosomiasis and strongyloidiasis. Appointment completion rates were also lower amongst children referred ‘out of area’. Additional potential barriers included vaccine hesitancy, needle phobia and interpretation challenges.

Referrals for TB and BBV at the ‘one‐stop’ clinic were 100% in line with national guidance and 96% for vaccinations—higher than in other studies [2, 7]. Attendance at the ‘one‐stop’ clinic for ID screening was 79%, suggesting effective internal processes and staff familiarity with the pathway. Missed appointments were mostly amongst children referred ‘out of area’, where logistical confusion contributed to nonattendance. These children either did not have their IHA at the ‘one‐stop’ clinic or their follow‐up appointments were not at the designated local acute hospital. Over half of those referred elsewhere missed appointments due to confusion about time and location.

Only 2/7 children who underwent their IHA at neighbouring trusts attended BBV testing, and 2/5 attended TB screening; both of the latter tested positive for latent TB. Although the sample size is small and the groups are not directly comparable, these findings suggest lower appointment completion rates amongst children seen out of area. Currently, these children are not eligible for the local ‘one‐stop’ clinic. Further research with a larger sample size is recommended to confirm these findings. If consistent, consideration should be given to enabling all UASC within this local authority to access both IHA and ID services through the ‘one‐stop’ clinic.

Sexual health referrals were not analysed for adherence due to the nuanced guidance, which emphasises child‐centred care and avoiding distress. Though underreporting may exist, only 13% were tested for chlamydia and gonorrhoea and 0% for syphilis. Miscommunication between services led to the assumption that syphilis was covered by another team. Recommendations include incorporating syphilis testing into the BBV screen and shifting chlamydia/gonorrhoea testing to the ID appointment. Practitioners should continue using discretion to avoid unnecessary distress.

Symptomatic presentation referrals were consistent with other studies (mainly skin and digestive conditions), and all were appropriately referred, demonstrating effective protocols. However, no patients were screened for schistosomiasis, strongyloidiasis or other soil‐transmitted helminths, despite RCPCH guidance recommending screening or empirical treatment for those from endemic countries. These should be offered at the ‘one‐stop’ clinic, or empirical treatment should be considered.

The median wait time from IHA to ID appointment was 4 months, with one delayed to 10 months due to an NHS number error. Delays like this are significant given the importance of early diagnosis and treatment, particularly for TB and BBVs. Pathways combining ID screening and IHA on the same day could reduce wait times and maximise engagement.

**Figure 8:**
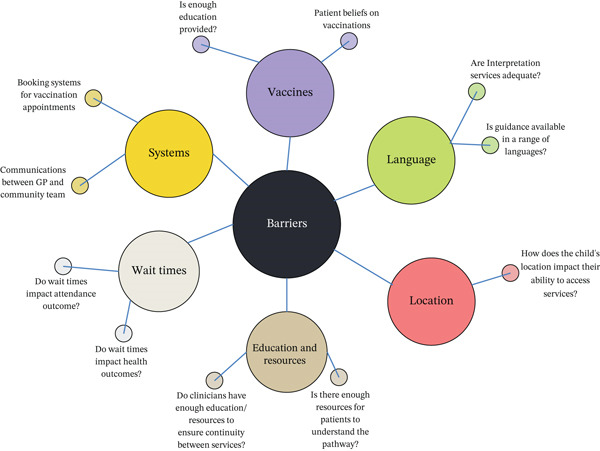
Scatter diagram of follow‐on project with hypotheses about which barriers UASC face when accessing local services.

Interpretation challenges affected 26% of cases, mostly at the GP level. In contrast, the IHA team used a consistent interpreter provider and did not experience these challenges. Interpreter protocol differences between services likely impact service access. Vaccine hesitancy or needle phobia was noted in 23%, though only one patient missed an appointment for this reason. These findings may inform future regional pathway development (see Figure 8).

The study captured data from all UASC under one department across a year, enabling specific service improvement recommendations. It also highlighted wider hypotheses around barriers to care. However, limitations include the small sample size, data limited to one local authority and reliance on retrospective documentation, which may under‐represent discussions or referrals. A prospective regional study would address these gaps (see Figure 8).

The lack of national comparative data limits benchmarking against other pathways. Cross‐sectional studies across multiple regions are needed to analyse referral, attendance and positive screening rates to improve national care consistency for UASC.

## 4. Recommendations

The recommendations following this project are two‐fold: (1) immediate considerations for local services as detailed above and (2) the initiation of further research to determine barriers to accessing ID services for UASC in the region, which may potentially be used to inform a regional ID pathway (see Figure 8). This research could delve deeper into the issues identified here, particularly focusing on language and interpretation challenges, vaccine hesitancy, needle phobia and the operational aspects of booking systems across all services, including vaccination appointments in primary care centres. The research would also use an open‐ended approach to analyse barriers that may not have been identified here. This would aim to facilitate a comprehensive understanding of the factors influencing UASC access to ID care services in the region. Combining findings from this study with the research conducted in this paper could result in a stronger pathway, possibly as a regional initiative. It has been shown that service standardisation for UASC (along with best practice guidance) can reduce the variation in practice and the consequent health disparities that may occur following this [8]. It is therefore worth exploring whether a regional pathway is feasible. This research could also inform further resources for both practitioners and patients. Improved guidance and education have been shown to improve both engagement and health outcomes for UASC [8]. This could manifest as a written pathway for clinicians across all ID services and as patient guidance (available in multiple languages) outlining the stages of their ID pathway.

## 5. Conclusions

This audit and retrospective analysis have demonstrated both positive and negative features of the current pathway for UASC under this local authority. On the one hand, referral and attendance rates were notably high compared with other single‐centre studies, showing particular success with the ‘one‐stop’ clinic at the local acute hospital. On the other hand, referrals and testing for some conditions such as syphilis, schistosomiasis and strongyloidiasis were overlooked, demonstrating interdepartmental miscommunication. Additionally, there was a trend in lower appointment attendance for children seen or referred ‘out of area’. In addition to several recommendations addressing immediate changes, it is further suggested that more research should be undertaken on the barriers faced by UASC when accessing ID services in the region, with the potential to inform a more robust, potentially county‐wide, pathway.

## Funding

No funding was received for this manuscript.

## Conflicts of Interest

The authors declare no conflicts of interest.

## Data Availability

The data that support the findings of this study are available from Dr Holly Tarn. Restrictions apply to the availability of these data, which were used under license for this study. Data are available from the authors with the permission of Dr Holly Tarn.
